# Artificial Intelligence in Orthopaedic and Trauma Surgery Education: Applications, Ethics, and Future Perspectives

**DOI:** 10.5435/JAAOSGlobal-D-25-00174

**Published:** 2025-09-10

**Authors:** Jaime Andrés Leal

**Affiliations:** From the Department of Orthopaedic Surgery, Hospital Universitario de La Samaritana, Bogotá, Colombia; Department of Medical Education, Universidad de la Sabana, Chía, Colombia; and Specialization in Artificial Intelligence, Pontificia Universidad Javeriana, Bogotá, Colombia

## Abstract

Artificial intelligence (AI) is redefining surgical education by enabling personalized, data-driven learning environments. In orthopaedic trauma surgery, a specialty defined by diagnostic complexity, time-sensitive decision making, and procedural precision, AI tools are uniquely positioned to enhance resident training. This narrative review explores the role of AI subfields—machine learning (machine learning), deep learning, computer vision, natural language processing, and generative AI—in orthopaedic education. Each technology supports distinct educational functions, from real-time performance tracking and image interpretation to examination simulation and feedback automation.

We describe how machine learning and deep learning models can assess technical competence and predict skill progression, whereas computer vision and augmented reality technologies provide immersive simulation and motion analysis. Natural language processing enables documentation analysis and scenario-based teaching, and large language models like ChatGPT support interactive, case-based learning. Ethical concerns such as algorithmic bias, data governance, transparency, and cognitive over-reliance are also discussed. A systems-based framework is proposed to integrate these technologies into a closed-loop educational cycle, emphasizing adaptive learning and professional growth.

AI is not a substitute for surgical mentorship, but a powerful amplifier of educational quality. Its thoughtful implementation can foster equity, efficiency, and innovation in orthopaedic trauma training—transforming how surgical competence is acquired, assessed, and advanced.

The advent of artificial intelligence (AI) has ushered in a paradigm shift in medical sciences, and its integration into surgical education is rapidly redefining traditional teaching models.^1^ Among all surgical specialties, orthopaedic trauma surgery is uniquely positioned to benefit from AI-enhanced training because of its heavy reliance on radiologic interpretation, procedural precision, real-time decision making, and the growing demand for simulation-based learning.

## Defining Artificial Intelligence and Its Subfields

AI is broadly defined as the ability of machines to perform tasks that typically require human intelligence, including reasoning, learning, decision making, and natural language understanding. Within AI, several subfields are directly relevant to surgical education.

1. Machine learning (ML): Algorithms that learn from structured data to make predictions or classifications without being explicitly programmed.^2^
2. Deep learning (DL): A subset of ML using neural networks with multiple hidden layers to model complex relationships in high-dimensional data, such as radiographs or surgical video.^3^
3. Computer vision (CV): The capacity of AI systems to interpret and act upon visual information such as medical images or live surgical footage.^4^
4. Natural language processing (NLP): Enables machines to understand, interpret, and generate human language, playing an increasing role in documentation analysis, examination preparation, and self-directed learning platforms.^5^
5. Generative AI: Emerging tools such as large language models (LLMs) that can create new, coherent text and simulate reasoning, increasingly evaluated for their educational applications.^6^

Each of these branches plays a different role in the educational continuum—from automating feedback to enabling real-time simulation, to enhancing radiological training, and even supporting ethical reasoning.

## Why Artificial Intelligence Matters in Orthopaedic Trauma Surgery Education

Orthopaedic trauma surgery combines time-sensitive, high-stakes decision making with technical dexterity and deep anatomic knowledge. Training in this discipline has traditionally relied on apprenticeship models, cadaveric dissection, supervised operating room exposure, and didactic lectures. However, this paradigm is increasingly challenged by constraints on duty hours, variability in case exposure, and the complexity of modern surgical care.

AI introduces the possibility of standardized, data-driven, personalized learning environments that adapt to the needs and performance of each trainee.

For instance:

1. ML algorithms can analyze performance metrics across thousands of simulation sessions to identify technical weaknesses.^2,7^
2. CV systems can detect hand movement inefficiencies or improper implant placement in real time.^1,7^
3. Generative models can simulate rare trauma cases or provide instant feedback on clinical reasoning.^6,8,9^

Importantly, these tools are not limited to simulation—they are being integrated into clinical workflows, operating rooms, and electronic health record systems. This convergence means that today's surgical residents must not only use AI tools but also understand their principles, biases, and limitations.

## Aims of This Narrative Review

This review aims to:

1. Define and explain the major AI subfields relevant to orthopaedic and trauma surgical education.
2. Illustrate current applications of AI tools in training, skill assessment, radiographic interpretation, and simulation.
3. Examine ethical concerns such as algorithmic bias, data governance, and autonomy in surgical decision making.
4. Explore future directions for research, integration, and curricular reform.
5. Propose a visual curricular model, integrating Bloom taxonomy and AI competency progression.

This review is not a technical manual on AI programming. Instead, it is a scientifically rigorous, clinically grounded exploration of how AI is shaping the next generation of orthopaedic trauma surgeons.

## Core Artificial Intelligence Concepts and Branches in Surgical Education

AI is not a monolithic tool but a constellation of computational disciplines, each with unique characteristics and applications. In surgical education, the most impactful AI subfields are ML, DL, CV, NLP, and generative AI. These technologies offer educators and learners tools for analyzing performance, automating feedback, simulating decision making, and personalizing the learning process.

Understanding these subfields in depth is essential not only for using AI-based educational tools but also for critically evaluating their validity, scope, and limitations.

**Figure 1:**
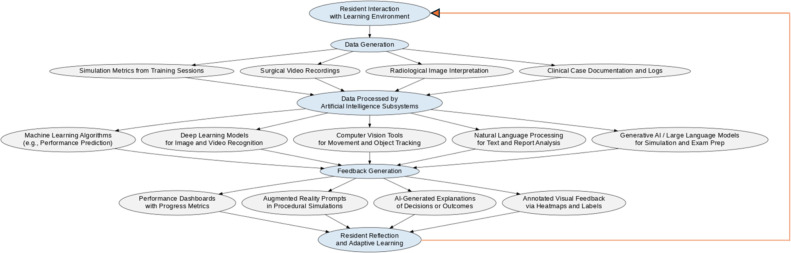
Workflow of artificial intelligence Integration in Orthopaedic Trauma Surgery Education. Diagram illustrating the cyclic process through which resident interaction with digital learning environments generates multimodal data. These data are processed by artificial intelligence subsystems—including machine learning, deep learning, computer vision, natural language processing, and large language models—to generate personalized feedback (eg, dashboards, augmented reality prompts, annotated explanations). This feedback informs reflective learning and adaptive strategies, completing a closed-loop educational system. Figure generated programmatically by the author using the Graphviz library within a Google Colab notebook (Python 3.11), based on a custom workflow model of artificial intelligence-assisted surgical education.

The integration of these AI subsystems into surgical education forms a closed-loop model of feedback and learning (Figure 1). This system captures how resident-generated data are processed by various AI branches to produce actionable insights and personalized educational feedback that enhances surgical competence.

### Machine Learning

Machine learning is a form of AI that enables systems to learn patterns from structured data and improve performance without explicit reprogramming. In surgical education, ML is frequently used for performance prediction, automated scoring, and curriculum personalization.^2,10,11^

ML algorithms are typically divided into:

1. Supervised learning: where the system is trained on labeled data (eg, motion trajectories labeled as “expert” or “novice”).
2. Unsupervised learning: where the model discovers hidden patterns in unlabeled data (eg, clustering resident surgical styles).
3. Reinforcement learning: where an agent learns by interacting with an environment and receiving feedback, potentially simulating real-time surgical decision making.^1,9^

In orthopaedics, supervised ML models have been used to classify surgical proficiency from motion sensor data collected during arthroscopy,^1,10^ predict resident progression based on simulator metrics,^12^ and even correlate radiological decision accuracy with learning curves.^13^ ML has also been used in adaptive educational platforms that recommend learning modules tailored to individual weaknesses.^8^

In trauma care, ML has proven useful in triage support, predictive scoring systems, and resource allocation models, which can also serve as scenarios for emergency simulation training.^14^

### Deep Learning

DL is a subcategory of ML that uses artificial neural networks with many layers to model complex relationships in data. DL models are particularly useful for analyzing unstructured data such as images, video, and audio—making them highly relevant for surgical education, especially in image interpretation and video-based skill assessment.

In orthopaedic training, DL has demonstrated effectiveness in:

1. Fracture detection, including subtle vertebral or femoral fractures.^3,15^
2. Phase recognition in surgical videos, allowing automated identification of procedural steps.^1,16^
3. Performance scoring, where resident videos are analyzed for errors in technique, pacing, or workflow.^1,10,17^

Recent studies show that DL models trained on cervical spine trauma CTs can match human experts in classifying complex fracture types,^3,15^ providing an opportunity for resident self-assessment during preoperative planning^15,18^.

### Computer Vision

Computer vision enables machines to interpret visual data and has gained ground in orthopaedics through applications in:

1. Radiograph and CT image interpretation.^3,19^
2. Tracking hand motion and tool usage in simulation environments.^20^
3. Anatomic landmark recognition, critical for real-time feedback in procedures like arthroscopy or pedicle screw placement.^17,21^

Some platforms now integrate augmented reality (AR) with CV to offer immersive training experiences. One such system has been validated in minimally invasive orthopaedic procedures, demonstrating improved anatomic understanding and spatial orientation among trainees.^12^

### Natural Language Processing

NLP enables machines to parse, analyze, and generate human language. In surgical education, NLP is used for:

1. Generating board-style examination questions from surgical texts.^22^
2. Analyzing reflective writing by residents for metacognitive skill assessment.
3. Real-time clinical decision support tools that explain rationale through natural language output.^23^

Emerging NLP systems can simulate full trauma bay interactions, generating patient histories, vital signs, and imaging reports while responding to resident questions. Their utility in teaching communication and prioritization is growing.^23^

### Generative Artificial Intelligence and Large Language Models

LLMs like ChatGPT can generate context-specific responses to surgical questions and case scenarios. Although their performance on OITE-style questions is promising,^6^ limitations such as factual hallucinations and lack of references necessitate cautious use.^24^

Some studies have also highlighted the risk of over-reliance on LLMs in surgical training, warning against using them for high-stakes decisions or without human oversight.^24^

Their best application lies in interactive learning, where they serve as Socratic agents prompting deeper reasoning, identifying logical gaps, or simulating case-based discussions.^23^

Recent improvements in LLMs—from GPT-3.5 to GPT-4o and 4.5—have reduced error rates and improved contextual understanding, yet performance still varies significantly across clinical domains and languages. Comparative analyses show that while newer models demonstrate higher accuracy in surgical knowledge recall and case interpretation, they continue to hallucinate references and generate plausible but incorrect rationales, particularly when challenged with rare cases or ambiguous scenarios.^19^ Continuous validation, transparency in updates, and educational supervision remain essential for their responsible deployment.

Table 1 summarizes the primary AI subfields applied in orthopaedic trauma education, highlighting their typical input data, core educational applications, and representative use cases.

**Table 1:** Core Artificial Intelligence Subfields in Orthopaedic Surgical Education: Input Modalities, Educational Roles, and Examples

AI Subfield	Input Data Types	Educational Applications	Representative Examples
Machine learning (ML)	Structured data (metrics, scores, logs)	Predictive modeling, adaptive feedback, resident progression analysis	Motion tracking to predict arthroscopy proficiency^10,20^
Deep learning (DL)	Images, video, 3D data	Fracture detection, phase recognition, skill scoring	Hip and cervical fracture classification from radiographs and CTs^3,4,15^
Computer vision (CV)	Video, real-time procedural footage	Tool tracking, anatomic landmark detection, AR feedback	AI-guided simulation with real-time alerts^12,16^
Natural language processing (NLP)	Surgical notes, examination texts, resident writing	Question generation, report feedback, reasoning analysis	Chatbot-based case teaching and automated documentation review^22^
Generative AI/LLMs	Free-text prompts and queries	Examination simulation, differential building, self-directed Q&A	ChatGPT answering OITE-style questions and generating explanations^6,24^

AR = augmented reality, LLMs = large language models, OITE = orthopaedic in-training examination

This summary outlines the input data types used by each AI branch, their specific applications in surgical training, and published examples of implementation within orthopaedic or trauma education contexts.

## Applications of Artificial Intelligence in Orthopaedic and Trauma Training

AI is revolutionizing how orthopaedic surgeons are trained, evaluated, and supported throughout their learning curve. Although many of these applications are still under validation, their implementation is steadily growing across academic institutions, residency programs, and surgical simulation centers.

### Automated Skill Assessment and Competency Tracking

One of the most mature applications of AI in surgical education is in automated performance assessment. Traditional surgical evaluation suffers from subjectivity and inconsistency, especially in technical skill appraisal.^2,4^ AI-powered systems can objectively analyze motion efficiency, tool usage, and procedural fluency across multiple modalities.

Some platforms use CV and ML to track:

1. Tool path lengths
2. Instrument collisions
3. Procedural step sequencing
4. Economy of motion^12,20^

Such systems generate automated feedback dashboards that can be integrated into portfolios for competency-based progression. Multicenter efforts are now underway to validate these systems across institutions, ensuring reliability and fairness in their application.^25^

### Diagnostic Imaging and Radiographic Interpretation

DL and CV have been particularly impactful in fracture detection, especially in hip, wrist, and vertebral trauma.^3,15^ Training platforms now combine thousands of AI-annotated radiographs into learning libraries, where residents can test diagnostic skills and compare against expert-level benchmarks.

Some systems provide interactive overlays that highlight regions of interest and contrast resident input with model predictions.^13^ These tools improve sensitivity and reduce interreader variability, especially among junior trainees.

Recent multicenter validations of convolutional neural networks for fracture detection show generalizability across training levels and image sources, suggesting that such tools are ready for broader educational deployment.^25^

### Procedural Guidance and Augmented Reality Simulation

CV-AR integration has enabled real-time anatomic overlays during simulation or cadaveric dissection, supporting safe technique development and spatial orientation.^12^ A validated AR simulation framework demonstrated significant improvements in accuracy and error reduction during minimally invasive orthopaedic procedures.^12^

In addition, intelligent simulators now incorporate AI to monitor resident actions and adjust feedback dynamically. These systems act as “virtual preceptors,” flagging unsafe techniques or suggesting ergonomic corrections during arthroscopic or open procedures.^5,21^

### Decision Making, Scenario-Based Simulation, and Chat-Based Tools

AI supports clinical decision-making training through interactive, scenario-based platforms that simulate trauma cases, evolving vitals, and patient dialog. These simulations help residents practice prioritization, triage, and surgical timing under stress.^14^

Models trained to replicate real-trauma triage decisions have shown high concordance with expert judgments and are now being adapted for simulation-based teaching in emergency scenarios.^14^

Chat-based tools such as ChatGPT are being explored for examination review, just-in-time learning, and case discussion simulations. Although they show promise in supporting differential diagnosis and knowledge recall,^6^ recent analyses highlight risks of inaccurate reasoning, overconfidence, and nonreferenced answers, emphasizing the need for human oversight.^24^

## Ethical Considerations and Recommendations in Artificial Intelligence–Based Surgical Education

The implementation of AI in orthopaedic education does not occur in a vacuum. While the potential benefits of AI are substantial, so too are the risks—especially when these technologies are integrated into high-stakes educational environments. Concerns over data privacy, algorithmic bias, lack of transparency, and the erosion of clinical autonomy have been widely reported in medical AI literature.^7,26^

### Data Privacy, Ownership, and Informed Consent

AI systems used in education depend on vast amounts of data—ranging from radiologic images and surgical videos to resident performance metrics and clinical documentation. Although these data provide the substrate for algorithm training, it often contain identifiable information from patients and residents alike.^7^

Informed consent protocols must clearly differentiate between:

1. Clinical use of data, such as using imaging to support diagnosis
2. Educational use, such as simulation-based feedback
3. Algorithm development, which may involve data sharing with third-party entities

Best practices recommend the use of tiered consent models, secure deidentification pipelines, and institutional data governance boards that review AI use cases before deployment.^7^

### Algorithmic Bias and Educational Equity

Bias is one of the most pressing issues in medical AI. When an algorithm is trained on data from a narrow demographic—such as predominantly adult male fracture cases—it may underperform in other populations, including women, children, or underrepresented ethnic groups.^13^

In educational settings, biased AI tools may:

1. Undervalue surgical variations or techniques
2. Penalize residents for nonstandard but safe approaches
3. Reinforce learning inequalities

Bias mitigation includes auditing across subgroups, data set diversification, and algorithm transparency.^7,13^

### Transparency, Explainability, and Trust

DL models are often “black boxes.” In surgical education, this raises key questions: Can residents understand their own AI-based scores? Can faculty interpret why an error was flagged?

Explainable AI techniques such as heatmaps, annotated timelines, or natural language explanations must become standard in educational tools.^13,25^

### Autonomy, Identity, and Over-Reliance

AI must support—not replace—clinical reasoning. Excessive dependence risks delaying professional identity formation and limiting cognitive growth. Recommendations include:

1. Sessions with AI guidance disabled
2. Structured reflection on AI suggestions
3. Resident empowerment in decision review^7,26^

### Institutional Responsibility

Academic centers bear the responsibility of validating tools, protecting data, and educating both faculty and trainees about AI systems. Accreditation agencies should begin setting minimum standards for AI use in education and assessment.^7,26^

### Global Access and Equity

The implementation of AI in surgical education must also confront disparities in technological infrastructure, data availability, and institutional readiness across global regions. In low- and middle-income countries, limited access to high-performance computing, proprietary software, or curated data sets can exacerbate educational inequities. Future frameworks must emphasize open-access platforms, cloud-based simulators, and international collaborations to ensure that AI-driven educational tools are inclusive, adaptable, and aligned with global training needs.^26^

## Future Perspectives and Challenges

The trajectory of AI in surgical education suggests not only continued expansion but also increasing integration and complexity. The question is no longer whether AI will become part of orthopaedic trauma training—it already is.^27^ The question is how to guide this transformation responsibly, efficiently, and equitably.

### Curricular Integration and Artificial Intelligence Literacy

Residency programs must incorporate formal AI education. Without this, trainees remain passive users of complex systems they do not understand. Suggested components include.

1. Basic ML/DL theory
2. Common sources of bias and error
3. Explainable AI methods
4. Critical appraisal of AI outputs^2,5,11^

**Figure 2:**
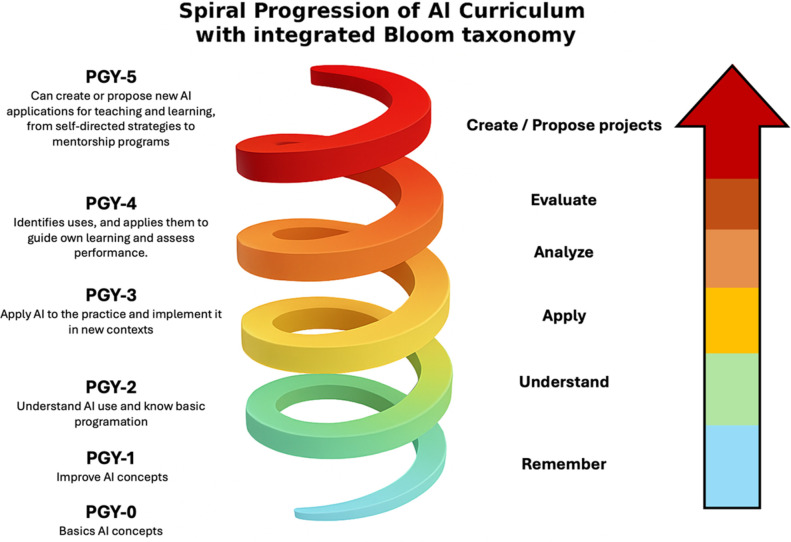
Spiral progression of an artificial intelligence curriculum in orthopaedic training aligned with Bloom's taxonomy. Diagram illustrating the sequential acquisition of artificial intelligence–related competencies across postgraduate years (PGY-0 to PGY-5), mapped to increasing levels of cognitive complexity. The spiral shape and color-coded segments represent both the progression and integration of skills, from foundational understanding to critical application and innovation. Figure generated using GPT-4o (open artificial intelligence) to render the 3D spiral, with all annotations, structure, arrow, and educational taxonomy integration added manually by the author in PowerPoint, based on a customized curriculum design framework for artificial intelligence in surgical education

Elective rotations or modules on AI in surgery are now emerging, but orthopaedics must adapt these to procedural and trauma contexts^18,23^ Table 2 and Figure 2.

**Table 2:** Artificial Intelligence Learning Objectives, Strategies, and Evaluation Methods by PGY Level

PGY Level	AI Learning Objectives	Educational Strategies	Evaluation Methods
PGY-1	Know basic ML/DL concepts and data types	Lectures, e-learning modules	MCQs, concept mapping
PGY-2	Understand basic AI programming and principles of application	Simulation laboratories, AI-assisted platforms	Performance dashboards
PGY-3	Apply AI tools in diagnostic and procedural simulations	Case-based discussion with AI outputs	OSCEs with AI feedback interpretation
PGY-4/5	Interpret AI-generated feedback and assess explainability Critically appraise AI models; integrate into OR planning	Mentored projects, journal clubs	Mini-CEX on AI-based decision support

PGY = postgraduate year, MCQs = multiple choice questions, OSCE = objective structured clinical examination, Mini-CEX = mini clinical evaluation exercise, OR = operating room

This outlines a staged curriculum framework for integrating AI literacy into orthopaedic and trauma surgery training, mapping core concepts to postgraduate years and competency milestones.

To complement the structured progression outlined in Table 2, this work proposes a visual curricular model that integrates Bloom taxonomy with a year-by-year trajectory of AI competencies. This 3D spiral representation (Figure 2) reflects not only the increasing cognitive complexity expected across residency years but also the expanding breadth of AI applications accessible at each stage of training. The spiral itself widens progressively, symbolizing how learners—initially operating within a narrow, guided scope—gain autonomy, versatility, and the capacity to integrate AI tools across broader clinical and educational contexts.

The accompanying vertical arrow, color-coded by Bloom taxonomy, adds an important dimension: The variable width of each cognitive level intentionally represents the perceived challenge and accessibility of those competencies. Early stages such as Understand or Apply appear broader, illustrating the greater cognitive effort required to incorporate AI tools that may initially feel foreign to established medical practice. As learners gain familiarity, subsequent levels become easier to assimilate—thus represented by narrower bands. Importantly, although reaching the Create level should remain an aspirational goal, it is acknowledged that not all orthopaedic surgeons must design novel AI systems; rather, they should be capable of critically proposing, adapting, or evaluating innovations within their scope of expertise.

Finally, the asymmetry in the spacing of the PGY stages along the spiral further reinforces this curricular logic: the early years (PGY-0 to PGY-2) are tightly grouped, reflecting foundational acquisition and limited application; by contrast, the upper stages (PGY-4 to PGY-5) open into wider arcs, symbolizing increased professional latitude and the multiplicity of directions in which AI can be implemented—ranging from clinical decision-making to personalized education or system-level transformation.

### Simulation, Mixed Reality, and Intelligent Tutoring

Immersive training environments using mixed reality, powered by CV and AI analytics, offer anatomic overlays, real-time alerts, and scenario branching logic.^12^ These systems allow.

1. Adaptation to resident skill level
2. Replay of past errors
3. Spatial reinforcement of anatomy^12,21^

As computational costs drop and cloud-based rendering improves, MR-AI fusion will likely become routine in simulation curricula.

### Predictive Analytics and Personalized Learning

ML models could track resident trajectories and predict outcomes such as:

1. Skill plateau
2. Simulation performance gaps
3. Readiness for OR autonomy

Such models, however, raise concerns about over-classification, feedback dependence, and labeling effects.^23^ AI should guide, not constrain, individualized growth.

### Multicenter Validation and Benchmarking

Many AI tools have been trained in narrow, single-center environments. Expanding to multicenter validation ensures generalizability and robustness.^25^

Hendrix et al^25^ demonstrated convolutional neural network fracture detection models maintained high accuracy across multiple trauma centers and imaging protocols, setting a precedent for educational model validation.

### International Frameworks Must Standardize

1. Training set reporting
2. Performance across learner subgroups
3. Grievance or override protocols in AI scoring systems^7^

### Augmented Mentorship and Continuing Education

AI will soon support life-long surgical learning. Imagine.

1. Continuous OR performance analysis
2. AI-flagged review cases
3. Personal dashboards for complication rates and remediation
4. Remote AI-facilitated mentorship in underserved regions^21^

Such systems must remain assistive, transparent, and open to human input—not prescriptive or punitive.^24^

### Critical Perspective on Generative Artificial Intelligence

Although generative models like ChatGPT show promise, recent analysis highlights key risks in surgical education.

1. Confidently incorrect answers (“hallucinations”)
2. Lack of references
3. Bias toward plausible-sounding but invalid logic^24^

Their value lies in guided self-assessment and Socratic prompting, not in decision authority.

### Financial Costs

Although the implementation of AI-based educational platforms may incur significant up-front costs—such as implant requirements, software licensing, and staff training—early reports suggest potential long-term savings through reduced faculty workload, improved resident autonomy, and more efficient remediation strategies. Cost-effectiveness analyses remain limited and represent a key area for future research.

## Summary

AI is no longer a future possibility in surgical education—it is an active agent of transformation. In orthopaedic and trauma surgery, where learning is rooted in procedural complexity, diagnostic precision, and time-critical decision making, AI offers tools that augment—not replace—traditional training.

Machine learning enables personalized education trajectories; DL powers radiographic and procedural analysis; CV provides real-time feedback and immersive simulation; NLP supports reasoning, documentation, and training simulations; and generative models promote self-directed, conversational learning.

Yet, none of these tools are neutral. Their ethical implementation demands transparency, fairness, autonomy, and validation. AI should serve not as a judge, but as a coach—an amplifier of surgical education rather than its arbiter.

The future of orthopaedic training will be hybrid, data-driven, and human-centered. Educators must take the lead—not just in adopting AI, but in shaping it to reflect the core values of surgical learning: critical thinking, equity, and professional growth.

Although developed within the context of orthopaedic trauma education, the principles and structural framework of this review may be adapted and extended across broader medical, surgical, and health professions education curricula.

## References

[1] Ward TM Mascagni P Madani A Padoy N Perretta S Hashimoto DA: Surgical data science and artificial intelligence for surgical education. J Surg Oncol 2021;124:221-230. 34245578 10.1002/jso.26496

[2] Laur O Wang B: Musculoskeletal trauma and artificial intelligence: Current trends and projections. Skeletal Radiol 2022;51:257-269. 34089338 10.1007/s00256-021-03824-6

[3] Kalmet PHS Sanduleanu S Primakov S, et al. Deep learning in fracture detection: A narrative review. Acta Orthopaedica. 2020;91:215. 31928116 10.1080/17453674.2019.1711323 PMC7144272

[4] Cha Y Kim JT Park CH Kim JW Lee SY Yoo JI. Artificial intelligence and machine learning on diagnosis and classification of hip fracture: Systematic review. J Orthop Surg Res. 2022;17:520. 36456982 10.1186/s13018-022-03408-7 PMC9714164

[5] Tian C Gao Y Rui C Qin S Shi L Rui Y. Artificial intelligence in orthopaedic trauma. EngMedicine. 2024;1.

[6] Hayes DS Foster BK Makar G, et al. Artificial Intelligence in Orthopaedics: Performance of ChatGPT on text and image questions on a complete AAOS orthopaedic in-training examination (OITE). J Surg Educ. 2024;81:1646. 10.1016/j.jsurg.2024.08.002 39284250

[7] Collins JW Marcus HJ Ghazi A, et al. Ethical Implications of AI in robotic surgical training: A Delphi consensus statement. Eur Urol Focus. 2022;613. 33941503 10.1016/j.euf.2021.04.006

[8] Lee B Nikhil N. Introduction to artificial intelligence for General Surgeons: A narrative review. Cureus. 2025;17. 10.7759/cureus.79871 PMC11958818 40171361

[9] De Simone B Abu-Zidan FM Gumbs AA, et al. Knowledge, attitude, and practice of artificial intelligence in emergency and trauma surgery, the ARIES project: an international web-based survey. World J Emerg Surg. 2022;17:10. 35144645 10.1186/s13017-022-00413-3 PMC8832812

[10] Vedula SS Ghazi A Collins JW, et al. Artificial intelligence methods and artificial intelligence-enabled metrics for surgical education: A multidisciplinary consensus. J Am Coll Surg. 2022;234:1181–1192. 35703817 10.1097/XCS.0000000000000190 PMC10634198

[11] Coppola A Hing C Asopa V, et al. Integrating artificial intelligence into trauma and orthopaedics: History, current state of AI in T&O and future perspectives. Orthopaedics Online. 2024.

[12] Cangelosi A Riberi G Titolo P, et al. Augmented reality simulation framework for minimally invasive orthopaedic surgery. Comput Biol Med. 2025;189. 10.1016/j.compbiomed.2025.109943 40088714

[13] Najjar R. Redefining radiology: A review of artificial intelligence integration in medical imaging. Diagnostics. 2023;13:2760. 37685300 10.3390/diagnostics13172760 PMC10487271

[14] Peng HT Siddiqui MM Rhind SG Zhang J da Luz LT Beckett A. Artificial intelligence and Machine Learning for Hemorrhagic Trauma Care. Military Medical Research. 2023;93. 10.1186/s40779-023-00444-0 PMC9933281 36793066

[15] Liawrungrueang W Cholamjiak W Promsri A, et al. Artificial intelligence for cervical spine fracture detection: A systematic review of diagnostic performance and clinical potential. Global Spine J. 2025;15:2547. 39800538 10.1177/21925682251314379 PMC11726500

[16] Baghdadi A Hussein AA Ahmed Y Cavuoto LA Guru KA. Computer vision technique for automated assesment of surgical performance using surgeonśconsole-feed videos. Int J Comput Assist Radiol Surg. 2019;14:697. 30460490 10.1007/s11548-018-1881-9

[17] Teatini A Kumar RP Elle OJ Wiig O: Mixed reality as a novel tool for diagnostic and surgical navigation in orthopaedics. Int J Comput Assist Radiol Surg 2021;16:407-414. 33555563 10.1007/s11548-020-02302-z PMC7946663

[18] Çalışkan SA Demir K Karaca O. Artificial intelligence in medical education curriculum: An e-Delphi study for competences. PLoS One. 2022;17:452. 10.1371/journal.pone.0271872 PMC9302857 35862401

[19] Huppertz MS Siepmann R Topp D, et al. Revolution or Risk?- Assesing the potential and challenges of GPT-4V in radiologic image interpretation. Eur Radiol; 2025. 10.1007/s00330-024-11115-6 PMC11836096 39422726

[20] Goldbraikh A Volk T Pugh CM Laufer S. Using open surgery simulation kinetic data for tool and gesture recognition. Int J Comput Assist Radiol Surg.; 2022:17. 10.1007/s11548-022-02615-1 PMC10766114 35419721

[21] De S Jackson CD Jones DB. Intelligent virtual operating room for enhancing nontechnical skills. JAMA Surg; 2023. 10.1001/jamasurg.2022.6721 PMC10753974 36920404

[22] Le KDR Tay SBP Choy KT, et al. Applications of natural language processing tools in the surgical journey. Front Surg. 2024;11. 10.3389/fsurg.2024.1403540 PMC11140056 38826809

[23] Leon S Lee S Perez JE Hashimoto DA. Artificial intelligence and the education of future surgeons. Am J Surg. 2025;246. 10.1016/j.amjsurg.2025.116257 39988540

[24] Lebhar MS Velazquez A Goza S Hoppe IC. Dr. ChatGPT: Utilizing Artificial Intelligence in Surgical Education. Cleft Palate Craniofac J. 2024;61:2067. 37545428 10.1177/10556656231193966

[25] Hendrix N Scholten E Vernhout B, et al. Development and validation of a convolutional neural network for automated detection of scaphoid fractures on conventional radiographs. Radiol Artif Intell. 2021;3. 10.1148/ryai.2021200260 PMC8329964 34350413

[26] De Simone B Chouillard E Gumbs AA Loftus TJ Kaafarani H Catena F. Artificial intelligence in surgery: The emergency surgeons´ perspective (the ARIES project). Discov Health Syst. 2022. 10.1007/s44250-022-00014-6 PMC9734362 37521114

[27] Lindsey R Daluiski A Chopra S, et al. Deep neural network improves fracture detection by clinicians. Proc Natl Acad Sci U S A. 2018;115. 10.1073/pnas.1806905115 PMC6233134 30348771

